# Inducible knockout of CHUK/IKKα in adult chondrocytes reduces progression of cartilage degradation in a surgical model of osteoarthritis

**DOI:** 10.1038/s41598-019-45334-5

**Published:** 2019-06-20

**Authors:** Kirsty L. Culley, Samantha G. Lessard, Jordan D. Green, Justin Quinn, Jun Chang, Tyler Khilnani, Elisabeth B. Wondimu, Cecilia L. Dragomir, Kenneth B. Marcu, Mary B. Goldring, Miguel Otero

**Affiliations:** 1 HSS Research Institute, Hospital for Special Surgery, New York, NY 10021 USA; 2 Weill Cornell Medical College, New York, NY 10021 USA; 3 Biochemistry and Cell Biology Department, Stony Brook University, Stony Brook, NY 11794 USA

**Keywords:** Osteoarthritis, Mechanisms of disease

## Abstract

CHUK/IKKα contributes to collagenase-driven extracellular matrix remodeling and chondrocyte hypertrophic differentiation *in vitro*, in a kinase-independent manner. These processes contribute to osteoarthritis (OA), where chondrocytes experience a phenotypic shift towards hypertrophy concomitant with abnormal matrix remodeling. Here we investigated the contribution of IKKα to OA *in vivo*. To this end, we induced specific IKKα knockout in adult chondrocytes in AcanCreER^T2/+^; IKKα^f/f^ mice treated with tamoxifen (cKO). Vehicle-treated littermates were used as wild type controls (WT). At 12 weeks of age, WT and cKO mice were subjected to the destabilization of medial meniscus (DMM) model of post-traumatic OA. The cKO mice showed reduced cartilage degradation and collagenase activity and fewer hypertrophy-like features at 12 weeks after DMM. Interestingly, in spite of the protection from structural articular cartilage damage, the postnatal growth plates of IKKα cKO mice after DMM displayed abnormal architecture and composition associated with increased chondrocyte apoptosis, which were not as evident in the articular chondrocytes of the same animals. Together, our results provide evidence of a novel *in vivo* functional role for IKKα in cartilage degradation in post-traumatic OA, and also suggest intrinsic, cell-autonomous effects of IKKα in chondrocytes that control chondrocyte phenotype and impact on cell survival, matrix homeostasis, and remodeling.

## Introduction

Articular chondrocytes are terminally differentiated, quiescent cells that display stable phenotypes functionally characterized by a slow matrix turnover, with a fine-tuned balance between anabolic and catabolic actions, which are responsible for maintaining the structural and functional integrity of articular cartilage^1^. This balance is lost in osteoarthritis (OA), a highly prevalent and disabling disease that represents a tremendous personal and socioeconomic burden^2^. OA affects and involves all joint tissues and especially impacts articular cartilage structural integrity, which undergoes irreversible destruction and leads to the need for total joint replacement surgery in patients with late-stage disease^1,3^.

In response to the abnormal biomechanical and biochemical environment of the OA joint, articular chondrocytes lose their phenotypic stability and acquire a hypertrophy-like phenotype^4^, displaying increased expression and activities of matrix-degrading enzymes, such as collagenase-3^5,6^, and abnormal production of matrix structural proteins, including type X collagen^7,8^. Ultimately, these phenotypic alterations result in perturbations of the extracellular matrix integrity and composition, and are believed to both initiate and perpetuate cartilage destruction. The phenotypic shift characteristic of OA is linked to the abnormal expression and activity of signaling molecules that drive chondrocyte hypertrophy, including Wnt^9^ and Notch^10^ signaling, Ihh^11^, Runx2^12^ and Hif2alpha^13,14^. Importantly, modulation of these pathways to inhibit chondrocyte hypertrophy modifies disease progression and maintains cartilage structural integrity^15–18^. Thus, a better understanding of the mechanisms that lead to loss of phenotypic stability in OA chondrocytes may help us to develop efficacious and targeted therapeutic strategies that prevent cartilage destruction.

Abnormal NF-κB signaling contributes to cartilage degradation^19,20^ and numerous studies have shown that NF-κB enables chondrocyte hypertrophy in OA (reviewed in^4,21^). We showed that CHUK/IKKα, which controls the non-canonical NF-κB pathway^21^, plays a predominant role in chondrocyte hypertrophy and matrix remodeling *in vitro*^22,23^ in a kinase-independent manner^23^. This is reminiscent of the requirement of the kinase- and NFκB-independent functions of IKKα in keratinocyte terminal differentiation^24–26^. However, the precise contribution and modes of action of IKKα in cartilage degradative processes *in vivo*, as well as its contribution to cartilage homeostasis and matrix turnover degradation in OA disease, were not addressed in previous studies. Here we show that mice with inducible and specific IKKα knockout in adult chondrocytes are protected from cartilage degradation after surgical induction of post-traumatic OA. We also show that IKKα has cell-intrinsic roles in adult articular and growth plate chondrocytes *in vivo*, which impact on cell survival, phenotypic stability, and matrix homeostasis and remodeling.

## Results

### Generation and characterization of cartilage-specific inducible IKKα knockout mice

**Figure 1:**
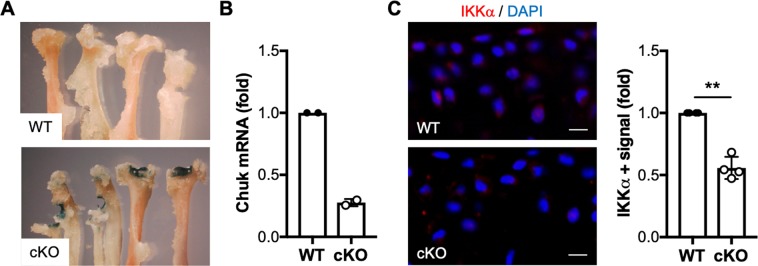
Characterization of mice with inducible cartilage-specific IKKα knockout. Joint tissues retrieved from 12-week-old AcanCreER^T2/+^; IKKα^f/f^ at 2 weeks after injection with vehicle (WT) or tamoxifen (cKO) were subjected to X-gal staining to assess tamoxifen-inducible cartilage-specific Cre recombinase activity. Representative images of the humerus, radius and ulna of the forelimbs obtained from WT and cKO mice are shown. (**A**) RTqPCR analyses in total RNA isolated from WT and cKO articular cartilage showed reduced IKKα mRNA in cKO samples (n = 2/ea) (**B**) and immunohistochemical analyses showed depletion of the IKKα protein in cKO articular cartilage (n = 4/ea). (**C**) Quantification of the positive immunostaining is shown on the left. Red = IKKα, Blue = DAPI. Scale bar = 10 μm **p = 0.0022 by one-sample *t*-test.

Previous studies showed that global IKKα-deficient mice display skin abnormalities concomitant with defective limb morphogenesis and perinatal lethality^27–29^; and thus, to study the role of IKKα in OA articular chondrocytes *in vivo*, we generated mice with inducible aggrecan-driven cartilage-specific IKKα knockout, where IKKα knockout can be induced in adult chondrocytes. The resulting AcanCreER^T2/+^; IKKα^f/f^ mice^30,31^ were born at the expected Mendelian ratio, with no gross abnormalities. These mice enabled us to induce IKKα knockout exclusively in aggrecan-expressing cells by tamoxifen treatment (cKO), and to use vehicle-treated littermates as wild type controls (WT). X-gal staining of tissues retrieved from mice injected with vehicle or tamoxifen showed specific tamoxifen-driven Cre-recombinase activity in cartilaginous tissues (Fig. 1A and Supplementary Fig. 1). RTqPCR analyses of total RNA isolated from articular cartilage (Fig. 1B) and immunostaining of IKKα in paraffin-embedded sections of WT and cKO mice (Fig. 1C) demonstrated efficient IKKα knockout in mice treated with tamoxifen. Additional RTqPCR analyses of total RNA isolated from WT and cKO cartilage at 8 weeks after surgical induction of OA further confirmed the reduced IKKα mRNA levels upon tamoxifen treatment (Supplementary Fig. 2). The lack of complete knockout when analyzing full-thickness articular cartilage was expected given previous reports showing that aggrecan-expressing cells (and therefore Cre-recombinase activity) are restricted to the upper cartilage layers in mice of this age^31^. Together, these analyses showed specific and efficient inducible knockout of IKKα in adult articular chondrocytes in mice that displayed no gross abnormality.

### Cartilage-specific IKKα KO mice show reduced osteoarthritis-like features following destabilization of the medial meniscus

**Figure 2:**
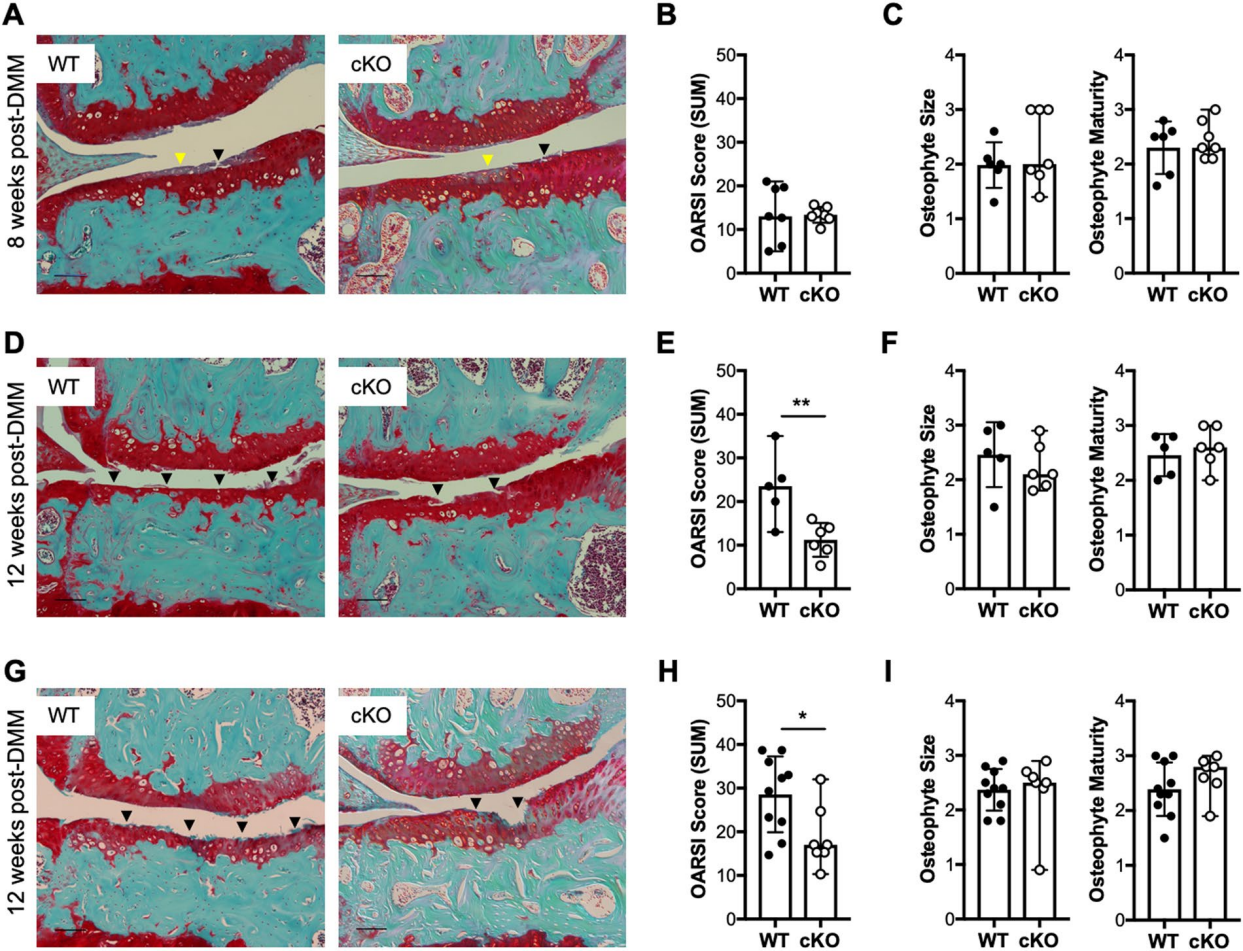
Mice with a cartilage-specific inducible IKKα knockout display attenuated cartilage degradation at 12 weeks after surgical induction of OA. Representative Safranin O/Fast green-stained sections and quantification of the cartilage degradation scores (shown as OARSI SUM scores) of the knee joints of DMM-operated 12-week-old male WT and cKO mice at (**A**,**B**) 8 weeks with KO induction before surgery (WT = 7; cKO = 7), (**D**,**E**) 12 weeks with KO induction before surgery (WT = 5; cKO = 6), and (**G**,**H**) 12 weeks with KO induction after surgery (WT = 10; cKO = 7). Images show the medial femorotibial compartment of DMM-operated knees using 10X magnification. Scale bar = 100 μm. Osteophyte size and maturity scores are shown in (**C**) for mice at 8 weeks post-DMM (WT = 6; cKO = 7), (**F**) for mice at 12 weeks post-DMM with KO induction before surgery (WT = 5; cKO = 6), and (**I**) for mice at 12 weeks post-DMM with KO induction after surgery (WT = 10; cKO = 7). Arrowheads indicate areas of proteoglycan depletion (yellow) and cartilage erosion (black). *p < 0.05, and **p < 0.01 by *t*-test.

Next, to determine whether IKKα contributes to cartilage remodeling and OA disease *in vivo*, we compared cartilage degradation in WT and cKO mice after surgical induction of OA. To do this, we first induced IKKα knockout at 2 weeks before surgery in 10-week-old AcanCreER^T2/+^; IKKα^f/f^ male mice. At two weeks after injection of tamoxifen or vehicle, we subjected the cKO and WT mice to DMM surgeries^32,33^ and we evaluated cartilage degradation^33,34^ at 8 and 12 weeks after surgery. Articular cartilage structural integrity did not differ between cKO and WT littermates in non-operated knees at 8 or 12 weeks after surgery (Supplementary Fig. 3). At 8 weeks post-DMM, we observed proteoglycan depletion accompanied by fibrillations and evident structural damage in both WT and cKO DMM-operated articular cartilage, with no significant difference in the OARSI scores between groups (Fig. 2A,B). However, at 12 weeks post-surgery the cKO mice showed significantly reduced cartilage damage compared to WT controls (Fig. 2D,E). To evaluate whether the structural cartilage protection at 12 weeks after surgery required IKKα knockout before disease initiation, we next induced IKKα knockout at 3 weeks after surgery, which had been performed in 12-week-old male AcanCreER^T2/+^; IKKα^f/f^ mice. Consistent with our previous result, the cKO displayed reduced cartilage degradation at 12 weeks after surgery compared to WT controls (Fig. 2G,H). We also analyzed the size and maturity of osteophytes, as described^33,35^. The non-operated, control knees did not form osteophytes, as expected, and we did not observe any difference in osteophyte formation, size, or maturity between groups at 8 or 12 weeks following surgery (Fig. 2C,F,I). These results indicate that cartilage-specific IKKα deletion impacts cartilage degradation without affecting osteophyte formation in a surgical model of post-traumatic OA.

### IKKα deficiency reduces collagenase activity and hypertrophy-like features in articular cartilage following destabilization of the medial meniscus

**Figure 3:**
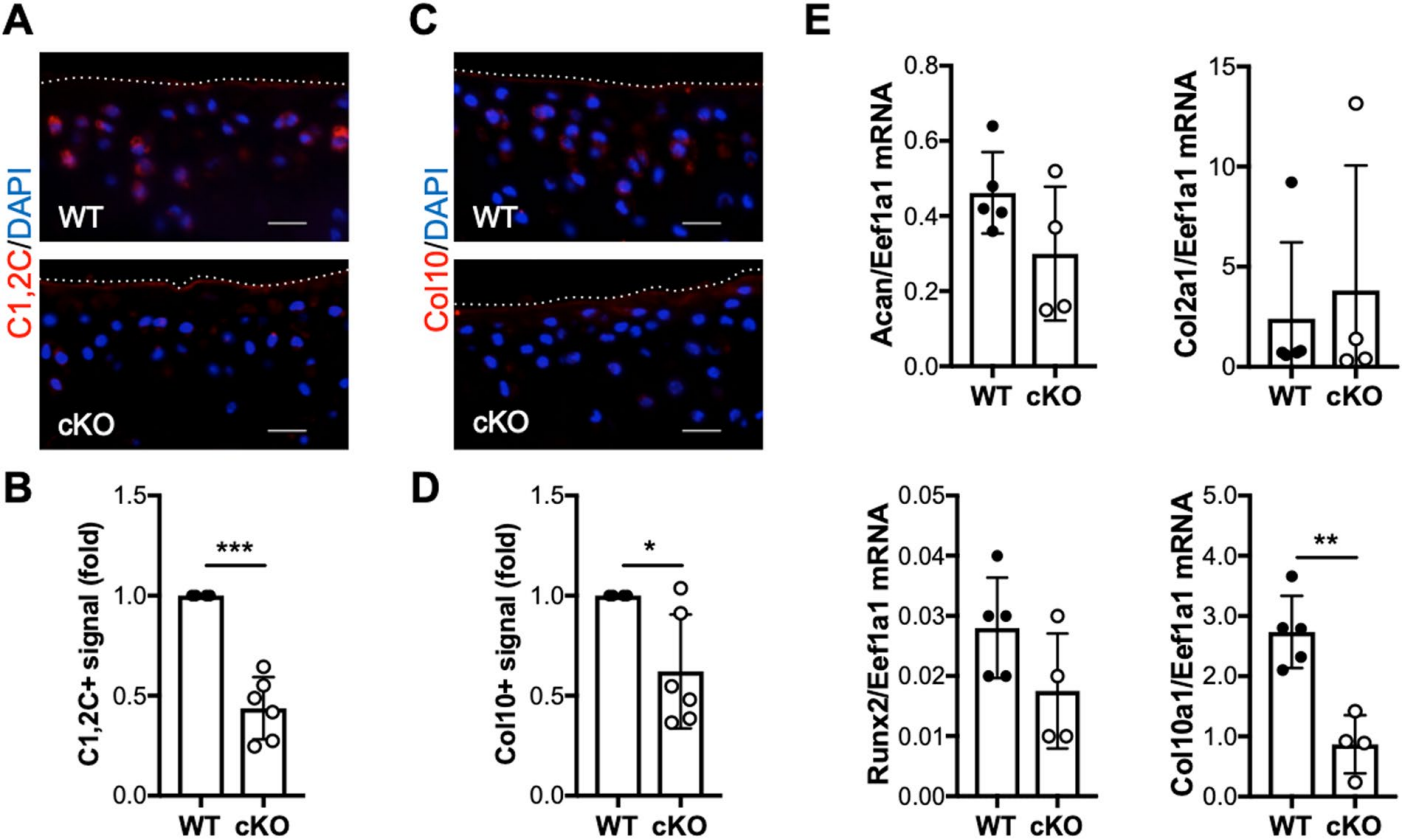
Reduced collagenase activity and hypertrophy in IKKα cKO cartilage following destabilization of the medial meniscus. Representative images and quantification of the positive immunostaining of sections from DMM-operated WT (n = 4) and cKO (n = 6) mice at 12 weeks post-surgery stained with specific antibodies against C1,2C (**A**,**B**) and type X collagen (Col10) (**C**,**D**). Red = C1,2C or Col10, Blue = DAPI. Scale bar = 20 μm. The dotted lines delineate the articular cartilage surface. *p = 0.0102 and ***p = 0.0003 by one-sample *t*-test. (**E**) RTqPCR analyses of Acan, Col2a1, Runx2 and Col10a1 mRNA were performed in total RNA isolated from WT (n = 5) and cKO (n = 4) articular cartilage at 8 weeks post-DMM. *p < 0.05, **p < 0.01, and ***p < 0.001 by *t*-test.

**Figure 4:**
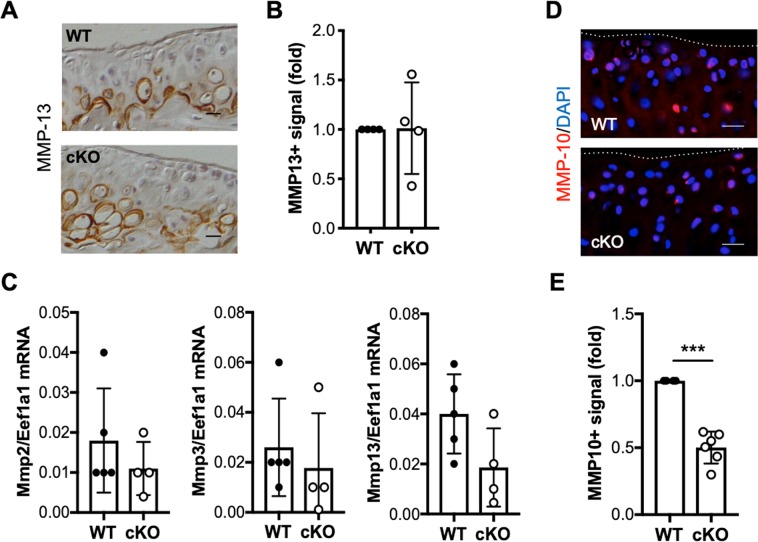
Decreased MMP-10 protein in IKKα cKO mice after surgical induction of OA. Representative images of WT and cKO sections at 12 weeks after DMM stained with specific antibodies against MMP-13 (**A**) and quantification of the MMP13-positive immunostaining (n = 4/ea). (**B**) RTqPCR analyses of Mmp2, Mmp3, and Mmp13 mRNA performed on total RNA isolated from WT (n = 5) and cKO (n = 4) cartilage at 8 weeks after DMM surgery. (**C**) Representative images of WT and cKO sections at 12 weeks after DMM stained with specific antibodies against MMP-10 (**D**), and quantification of the MMP10-positive immunostaining in WT (n = 4) and cKO (n = 6) samples. (**E**) Red = MMP-10, Blue = DAPI. The dotted lines delineate the articular cartilage surface. Scale bar = 20 μm. ***p = 0.0002 by one-sample *t*-test.

Human and murine IKKα-deficient primary chondrocytes display reduced collagenase activity and hypertrophic features *in vitro*^22,23^. To assess whether the cartilage protection observed in cKO mice post-DMM also correlated with decreased collagenase activity *in vivo*, we performed immunohistochemical analyses in WT and cKO cartilage sections using the C1, 2C antibody, which detects collagenase-specific cleavage epitopes in type II collagen^6^. Immunohistochemical analyses showed reduced C1, 2C immunostaining in cKO mice compared to WT counterparts at 12 weeks following DMM (Fig. 3A,B), in agreement with our previous results^22,23^. In keeping with our *in vitro* observations, type X collagen immunostaining was also reduced in the cKO cartilage samples at 12 weeks post-DMM (Fig. 3C,D), suggesting that hypertrophy-like differentiation was impaired in the absence of IKKα *in vivo*. This result was confirmed in total RNA isolated from control and DMM-operated WT and cKO mice at 8 weeks after surgery. RTqPCR analyses showed significantly decreased Col10a1 expression in cKO samples, whereas Acan and Col2a1 expression did not change between groups (Fig. 3E). We also observed an overall decrease in Runx2 mRNA in cKO cartilage, but the differences between WT and cKO samples were not significant due to the high variability (Fig. 3E). Matrix metalloproteinase-13 (Mmp13), or collagenase-3, is the main cartilage-degrading collagenase^6^. Mmp13 overexpression leads to OA-like features^36^, and Mmp13 deficiency has been linked to cartilage structural protection in OA models^35,37^ and to defective hypertrophy in growth plate chondrocytes^38,39^. Thus, we next assessed whether the reduced collagenase activity in cKO cartilage correlated with reduced levels of MMP-13 after DMM. Immunohistochemical analyses showed no difference in MMP-13 immunostaining between WT and cKO samples (Fig. 4A,B). RTqPCR analyses performed in total RNA extracted from cartilage samples showed that the Mmp13 mRNA levels were not significantly different between groups (Fig. 4C). The mRNA levels of other cartilage-degrading metalloproteinases, Mmp2 and Mmp3, were also similar in WT and cKO samples (Fig. 4C). Given that the levels of these MMPs remained unchanged, but the collagenase activity was reduced in the cKO cartilage, we next assessed whether IKKα deficiency was leading to changes in pro-collagenase activators. We previously reported that Mmp10 mRNA and encoded protein were reduced *in vitro*, in IKKα-deficient human and murine chondrocytes^23^. Indeed, our *in vivo* analyses in cKO mice showed that, while Mmp10 mRNA was not reliably detected using our conditions, the MMP-10 protein levels (Fig. 4D,E) were reduced significantly in IKKα-deficient cartilage at 12 weeks after DMM. These results are in agreement with our previous findings^22,23^ and suggest that the decreased collagenase activity in IKKα-deficient chondrocytes *in vivo* is due, at least in part, to the decreased levels of the pro-collagenase activator MMP-10^40^. Together, our results suggest that IKKα has cell-intrinsic roles in articular chondrocytes *in vivo*, and indicate that the IKKα cKO mice are protected against DMM-induced cartilage degradation and display reduced collagenase activity and hypertrophic-like features *in vivo*.

### Normal IKKα function is required for growth plate homeostasis

**Figure 5:**
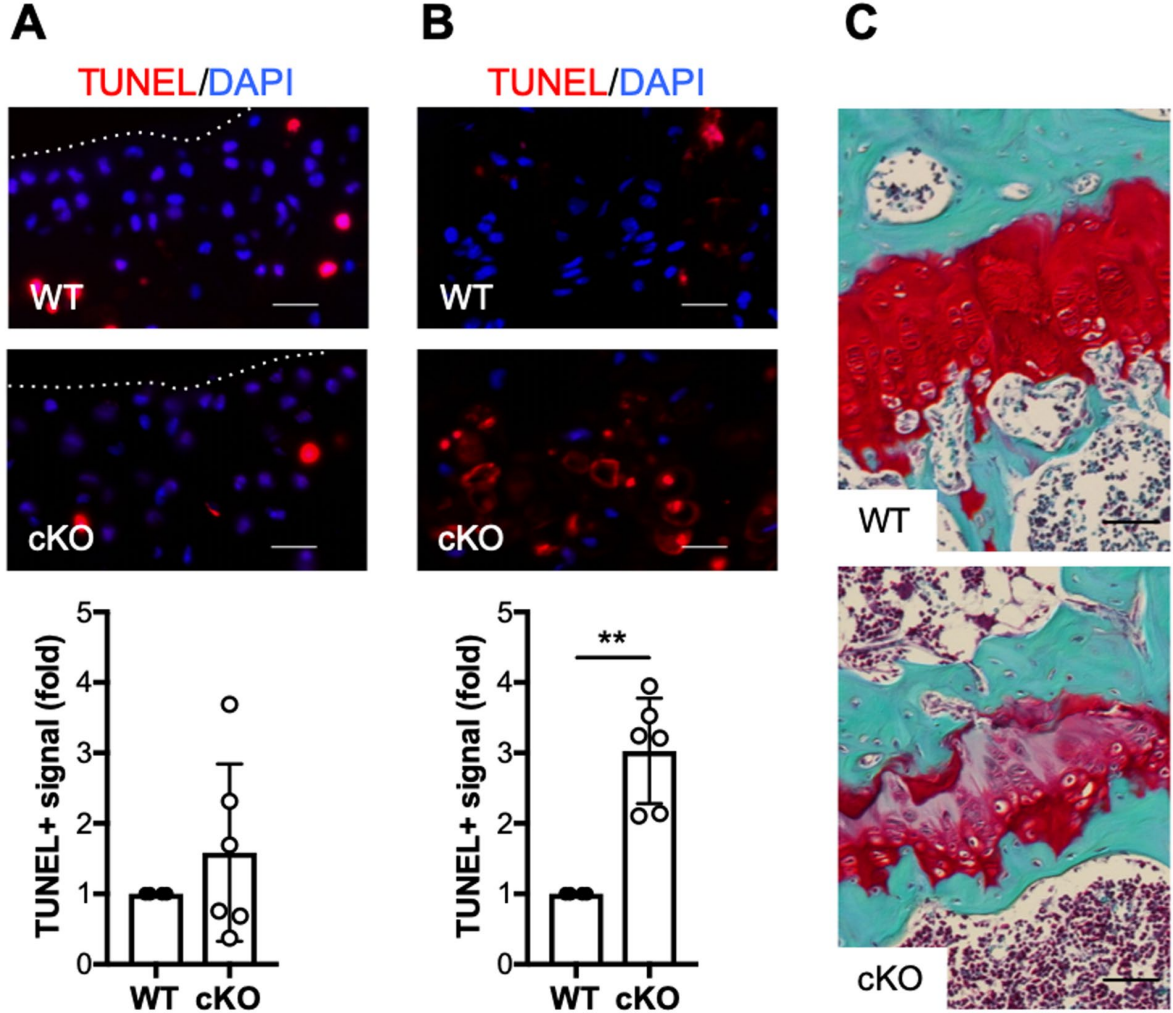
Abnormal post-natal growth plate architecture and increased chondrocyte apoptosis in IKKα cKO mice. (**A**) Representative TUNEL staining in articular cartilage of DMM-operated WT (n = 4) and cKO (n = 6) sections at 12 weeks after DMM surgery, and quantification of the TUNEL-positive signal. Scale bar = 20 μm. Red = TUNEL, Blue = DAPI. The dotted lines delineate the articular cartilage surface. (**B**) Representative TUNEL staining in growth plates of the same WT (n = 4) and cKO (n = 6) sections, and quantification of the TUNEL-positive signal. Scale bar = 20 μm. Red = TUNEL, Blue = DAPI. (**C**) Representative histological sections (Safranin O/Fast green stained) of growth plates of the same WT and cKO mice. Scale bar = 50 μm. **p = 0.0012 by one-sample *t*-test.

To further understand the cell-intrinsic roles of IKKα in chondrocytes with functional relevance to OA, we next evaluated its impact on cell viability by TUNEL in samples harvested at 12 weeks post-DMM. Given the structural protection observed in cKO cartilage post-DMM surgery, we were expecting higher cell death in WT mice. However, we observed a trend towards increased cell death in cKO cartilage samples, although the differences were not significant due to the high variability (Fig. 5A). When we assessed the number of TUNEL-positive (TUNEL+) chondrocytes in the growth plates of the same mice, we observed significantly increased numbers of dead cells in cKO limbs, as shown in Fig. 5B. Additional analyses revealed severe alterations of the growth plate architecture in 60% of the cKO mice (Fig. 5C), which display abnormal cellular arrangement and reduced proteoglycan content, assessed by Safranin-O staining.

Taken together, our results further suggest chondrocyte-autonomous effects of IKKα in adult articular and growth plate chondrocytes *in vivo*, which control cellular phenotypes and impact on cell survival and matrix homeostasis and remodeling.

## Discussion

Articular chondrocytes respond to the abnormal joint environment in OA disease by undergoing phenotypic modulation, catabolic activation, and hypertrophic differentiation^4^. These series of events lead to abnormal production of matrix structural proteins^7,8^ and irreversible destruction of the extracellular matrix that confers functional properties to articular cartilage^1,3^. Thus, the loss of phenotypic stability of articular chondrocytes is believed to be an early event with a central role in the initiation and progression of cartilage destruction in OA^4^. Our previous work showing that IKKα contributes to chondrocyte hypertrophy and matrix remodeling *in vitro*^22,23^ suggested its contribution to the loss of phenotypic stability and abnormal cartilage remodeling in OA disease. Here, we show that IKKα contributes to articular cartilage destruction in a model of post-traumatic OA. Our data also suggest that IKKα impacts articular chondrocyte hypertrophy and displays cell-intrinsic roles in both articular and postnatal growth plate chondrocytes *in vivo*.

We assessed the contribution of IKKα to cartilage remodeling and chondrocyte hypertrophy in OA *in vivo* using mice with inducible knockout in adult aggrecan-expressing tissues^31^, which allowed us to bypass potential confounding defects driven by knocking out IKKα during development. We initially subjected WT and cKO male AcanCreER^T2/+^; IKKα^f/f^ mice to the DMM model^32,33^ at 2 weeks after vehicle or tamoxifen administration. While cartilage degradation scores were identical between WT and cKO at 8 weeks post-DMM, the cKO showed significantly reduced cartilage degradation at 12 weeks after surgery. The overall cartilage degradation did not progress in DMM-operated cKO cartilage between 8 and 12 weeks (p = 0.5985) while it increased significantly in the WT groups (p = 0.0321). These results suggested that IKKα could be a contributing factor to the progression phase of the disease. To test whether the structural protection was similarly observed if IKKα knockout was induced after surgical induction of OA, we also subjected AcanCreER^T2/+^; IKKα^f/f^ mice to DMM surgeries and induced IKKα knockout at 3 weeks after surgery. Consistent with our results knocking out IKKα before DMM, these cKO mice showed decreased cartilage degradation scores at 12 weeks after surgery. Thus, taken together our findings suggested that IKKα impacts the progression phase of DMM-induced OA disease. Moreover, the reduction in cartilage degradation was independent of osteophyte formation, size and maturity, which were identical in WT and cKO mice.

In addition to structural protection, our data suggest that the cKO mice displayed reduced hypertrophy-like features after DMM, as indicated by the reduced levels of type X collagen mRNA and protein in operated knees. Changes in type X collagen were accompanied by decreased collagenase activity, similar to the findings in other mouse models with pharmacological or genetic modifications of factors involved in hypertrophic differentiation^5,10–18^. Chondrocyte hypertrophy is a developmental process required for endochondral ossification whereby growth plate chondrocytes undergo a synchronized series of events that involve phenotypic differentiation, matrix deposition, calcification, increased collagenase activity, and apoptosis^41^. MMP-13 is required for growth plate development, where it is synthesized by hypertrophic chondrocytes^38,39^, and it is the main collagenase in OA cartilage^6^, where it is produced by OA articular chondrocytes and contributes to cartilage degradation^35–37^. Thus, it follows that impaired chondrocyte hypertrophy in OA models is accompanied by reduced MMP-13 levels and subsequently decreased collagenase activity^12–14,42^. However, while type X collagen and collagenase activity were reduced in IKKα cKO mice post-DMM, the Mmp13 mRNA and encoded protein levels were not significantly changed. The expression levels of other cartilage-degrading MMPs were similarly unchanged in WT and cKO cartilage after surgery, suggesting that the activity of MMP-13 and not the level of the protein is modulated by IKKα or its downstream effectors *in vivo*. MMP-10 is a stromelysin that can act as a pro-collagenase activator with functional implications in articular cartilage remodeling^40^, and we reported previously that Mmp10 mRNA and protein levels are reduced in IKKα-deficient chondrocytes *in vitro*, independent of the kinase activity of IKKα^23^. Similarly, our *in vivo* evaluation of WT and cKO cartilage following DMM revealed that, indeed, MMP-10 protein levels were reduced in IKKα cKO mice. The reduction in MMP-10 protein could help to explain, at least in part, the reduced collagenase activity independent of the relative gene and protein expression of the MMPs analyzed. To note, using our conditions we did not reliably detect Mmp10 mRNA in RNA extracts from microdissected articular cartilage after DMM surgeries. Therefore, whether changes in IKKα activity directly drive Mmp10 expression *in vivo*, or whether the changes observed in MMP-10 protein levels are driven by indirect mechanisms, could not be determined in this study.

Chondrocyte apoptosis has been linked to OA disease, and both intrinsic and extrinsic apoptotic mechanisms have been observed in human OA cartilage and in mouse models of OA^43^. Thus, given the structural protection in cKO mice after DMM, we explored whether IKKα activity was also contributing to the survival of articular chondrocyte in this model of post-traumatic OA. While we did not detect significant differences in TUNEL+ cells comparing WT and cKO articular cartilage at 12 weeks after surgery, we found significantly increased chondrocyte apoptosis in the IKKα cKO growth plates of the same mice. In addition, the growth plates of these cKO mice displayed abnormal architecture and composition, with disorganized cellular arrangement and decreased proteoglycan content. Given that these structural abnormalities were not observed in the articular cartilage of the same animals upon histological examination, it is conceivable that these effects of IKKα in adult growth plate chondrocyte homeostasis relate with the more dynamic nature of growth plate chondrocytes relative to the quiescent articular chondrocytes^44^, and their requirement for a more rapid and synchronized matrix turnover. Previous reports using global IKKα-deficient mice suggested that the contribution of IKKα to growth plate chondrocytes and abnormal limb morphogenesis was driven by the actions of the protein in keratinocytes^25^. However, these studies did not address specifically whether IKKα had chondrocyte-intrinsic actions, independent of its impact on keratinocytes. Our results in adult articular cartilage and postnatal growth plates following DMM surgery suggest that IKKα displays chondrocyte-intrinsic roles that may affect viability and matrix remodeling *in vivo*. Therefore, the specific contribution and modes of action of IKKα in growth plate development and maintenance merit further investigation.

Together, our *in vivo* findings suggest that IKKα is a critical regulator of cartilage remodeling and chondrocyte hypertrophy *in vivo*. While the small sample size and lack of sham controls are limitations of this study, the results here presented agree with our *in vitro* observations^22,23^ and highlight the importance of IKKα to cartilage homeostasis *in vivo*. However, a better understanding of the roles and mechanisms of action of IKKα in cartilage is still required. For instance, additional modes of IKKα-dependent control of collagenase activity could be at play in the absence of IKKα in chondrocytes, including changes in the levels of tissue inhibitors of metalloproteinases such as Timp3^23^, not explored in this study. In addition, the mRNA and protein levels of the collagenases evaluated in this study were assessed at the same or proximate time point(s) as the byproducts of collagenase activity and, therefore, our results do not rule out that IKKα deficiency could lead to decreased mRNA or protein at earlier times *in vivo*. Similarly, concomitant changes in articular cartilage and growth plates should be further addressed to better understand whether structural changes in the growth plate have functional impact and modify the progression of OA in articular cartilage. Furthermore, we have not determined whether these mechanisms of action of IKKα in adult articular and growth plate chondrocytes *in vivo* are dependent on NF-κB and/or kinase activity or occur primarily in the cytoplasm or nucleus. We also have not examined whether IKKα is engaging with signaling cascades other than NF-κB with functional relevance to cartilage homeostasis and degradation^21^. Understanding these mechanisms and other potential modes of action could help us to better define the downstream targets and interacting factors of IKKα, and could provide valuable insights into disease mechanisms and potential therapeutic interventions.

## Methods

See Supplementary Information for a detailed outline of the methods, procedures and specific materials used in this study.

### Ethics statement

All experiments were performed according to the guidelines of the American Veterinary Association and were approved by the IACUC of the Hospital for Special Surgery, and all procedures are reported following the ARRIVE guidelines^45^.

### Cartilage-specific inducible IKKα knockout mice

We generated mice with tamoxifen-inducible aggrecan (Acan)-driven deletion of IKKα by crossing AcanCreER^T2/T2^ knock-in mice^31^, which we originally obtained from Dr. Benoit de Chrombrughe’s lab (available from Jackson labs as B6.Cg-*Acan*^*tm1(cre/ERT2)Crm*^/J; stock# 019148) with existing IKKα^f/f^; R26R mice, harboring IKKα alleles flanked by LoxP recombination sites and carrying the R26R (Cre-dependent LacZ) reporter gene^30^. The resulting AcanCreER^T2/T2^; IKKα^f/f^:R26R mice were born at the expected Mendelian ratio, without gross abnormalities. All mice used for experiments were heterozygous for AcanCreERT2 (AcanCreER^T2/+^). For experimental purposes, male AcanCreER^T2/+^; IKKα^f/f^ mice were injected three times intraperitoneally with tamoxifen (2 mg per 10 g of body weight emulsified in sunflower oil) with two-day intervals to activate Cre-recombinase in aggrecan-expressing chondrocytes (experimental group, cKO). Vehicle-treated AcanCreER^T2/+^; IKKα^f/f^ mice were used as wild type controls (control group, WT). Tamoxifen was administered either to 10-week-old mice at 2 weeks before surgical induction of OA, allowing 1 week to recover from injections before surgery, or to 15-week-old mice at 3 weeks after surgery.

### Destabilization of the medial meniscus (DMM) surgery and tissue processing post-DMM

DMM surgeries were performed, as described^32,33^, in 12-week-old male IKKα cKO and WT littermates. Power analyses were performed to calculate sample size for the surgical experiments. The left knees were unoperated and served as contralateral controls. Animals were euthanized at 8 (WT = 7, cKO = 7) and 12 (WT = 5, cKO = 6) weeks post-surgery in experiments where IKKα knockout was induced before surgical induction of OA, or at 12 weeks post-surgery where IKKα knockout was induced after surgical induction of OA (WT = 10, cKO = 7). Knees were collected and processed for histological scoring of OA pathology^33^ assessed by two blinded scorers, as described^33,34^. Osteophyte size and maturity were evaluated in the same sections, also as described^33,35^. Briefly, coronal sections of 6 μm were cut across the whole joint, deparaffinized in xylene, rehydrated through an ethanol series, and stained with Safranin O/Fast green. OARSI SUM scores were obtained by grading 8 sections per knee joint (each data point represents the average of 8 sections/mouse). Similarly, osteophyte size and maturity scores represent the average of the scoring of 8 sections per mouse.

### Statistical analysis

Statistical analyses were performed using GraphPad Prism 7 Software (GraphPad Software, Sand Diego, CA). Data are reported as means ± S.D. or as median and 95% C.I. (histological scores) of at least three independent experiments. Unpaired Student *t*-test was used to establish statistical significance between two groups. One sample *t*-test analyses were performed for data represented as fold-changes, as indicated. P < 0.05 was considered significant.

## Supplementary information

Supplementary Information

## Data Availability

The data that supports the findings of this study are available from the corresponding author on reasonable request.
