# Supplementary material for: Inducible knockout of CHUK/IKKα in adult chondrocytes reduces progression of cartilage degradation in a surgical model of osteoarthritis
Source: Sci Rep. 2019 Jun 20;9:8905. doi: 10.1038/s41598-019-45334-5 (PMC6586628; doi:10.1038/s41598-019-45334-5)

SUPPLEMENTARY INFORMATION

Inducible knockout of CHUK/IKKα in adult chondrocytes reduces progression of cartilage degradation in a surgical model of osteoarthritis.

Kirsty L. Culley[1], Samantha G. Lessard[1], Jordan D. Green[1], Justin Quinn[1], Jun Chang[1], Tyler Khilnani[1], Elisabeth B. Wondimu[1,2], Cecilia L. Dragomir[1], Kenneth B. Marcu[3,4], Mary B. Goldring[1,2,4], and Miguel Otero[1*]

From the [1]HSS Research Institute, Hospital for Special Surgery, New York, NY 10021, USA; the [2]Weill Cornell Medical College, New York, NY 10021, USA; and the [3]Biochemistry and Cell Biology Department, Stony Brook University, Stony Brook, NY 11794, USA.

[*]To whom correspondence should be addressed: Miguel Otero, Ph.D., Hospital for Special Surgery, HSS Research Institute, Room 603, 515 East 71st Street, New York, NY 10021, USA; Tel. 212-774-7561; Fax. 617-249-2373; E-mail: OteroM@hss.edu

[4]K.B.M and M.B.G contributed equally to this work

Funding: This work was supported by National Institutes of Health Grants RC4 AR060546 (to M.B.G. and K.B.M.), AG022021 (to M.B.G.), and R21 AG049980 (to M.O.). J.D.G. received support from R01 AR066635.

X-gal staining:

Specific induction of Cre-recombinase activity was assessed in tissues harvested from tamoxifen- (cKO) and vehicle- (WT) injected mice subjected to X-gal staining. Briefly, tissues were harvested and fixed in a 4% paraformaldehyde solution in PBS for 1 hour at 4°C, washed for 15 minutes in PBS at room temperature, and incubated with X-gal substrate solution overnight at 37°C. After overnight incubation, the tissues were washed in PBS to remove excess stain and imaged to identify the areas of Cre-recombinase (LacZ-positive) activity.

Total RNA isolation:

We extracted total RNA from the articular cartilage of non-operated or DMM-operated mice, as described[1]. Briefly, articular cartilage was removed from the tibial plateaus and femoral chondyles with a scalpel blade while bathed in RNAlater (Ambion) under a dissection microscope. After removing RNAlater, articular cartilage samples were homogenized in TRIzol (Invitrogen) and total RNA was extracted using a modified protocol of the miRVana mRNA isolation kit (Ambion), also as described[1]. RNA integrity was assessed at the Core Laboratories Center of Weill Cornell Medicine. For RTqPCR analyses we used 100ng of total RNA with RIN from 6.8 to 9.9.

Real-time quantitative polymerase chain reactions (RT-qPCR)

Gene amplifications were carried out using SYBR Green I-based real-time PCR, as described[2] using specific primers against *Chuk*, *Mmp2*, *Mmp3*, *Mmp13*, *Mmp10*, *Runx2, Col10a1*, *Col2a1* and *Acan* (**Supplementary Table 1**). The data were calculated as the ratio of each gene to *Eef1a1*, using the $2^{-\Delta\Delta Ct}$ method for relative quantification[3]. *Actb* was used as an additional housekeeping gene in control experiments, but not used as a normalizer in the final analyses.

Immunofluoresce (IF-P) staining and analyses:

Sections from WT and cKO joints at 12 weeks post-DMM were deparaffinized in xylene, rehydrated in an ethanol series, and incubated with 2 mg/ml hyaluronidase (Sigma-Aldrich) for 30 min at 37°C. Adjacent sections were incubated with specific antibodies against IKKα (Santa Cruz), collagen X (Col10, Abcam), C1,2C (IBEX), or MMP-10 (Santa Cruz) overnight at 4°C. The sections were then incubated with AlexaFluor555 conjugated secondary antibodies (Cell Signaling) for 2h at room temperature and mounted using ProLong Gold antifade medium with DAPI (Life Technologies). Control sections were incubated with isotype-matched IgG instead of the primary antibody (not shown). Relative quantification was performed basically as described[2].

Briefly, images were captured using a Nikon Eclipse Ni-E microscope, and the IKKα, Col10, C1,2C and MMP10-positive mean pixel density was measured and normalized to the DAPI+ signal. Signal levels were set to a threshold level based on isotype control staining.

MMP-13 immunohistochemistry (IHC) and analysis:

To assess differences in MMP-13 protein levels, immunostaining was performed in WT and cKO samples collected at 12 weeks post-DMM (n=4/ea) using the Vectastain ABC rabbit IgG kit (Vector Laboratories). Briefly, sections were deparaffinized in xylene and rehydrated in an ethanol series. After antigen retrieval (2 mg/ml hyaluronidase for 30 min at 37°C), quenching of endogenous peroxidases (3% hydrogen peroxide in PBS for 30 min at room temperature) and blocking, sections were incubated overnight with a rabbit polyclonal MMP-13 antibody (Abcam). The signal was developed using NovaRED (Vector Laboratories). For negative controls, normal rabbit IgG (Santa Cruz) was used in place of the primary antibody (not shown). Digital images of the MMP13-stained sections were obtained using a Nikon Eclipse Ni-E microscope (Nikon Instruments) with a DS12 bright-field camera and analyzed as described[4,5]. The total area of the region of interest (ROI) was measured in pixels. We selected 4 to 5 ROIs per microphotograph to cover the entire cartilage surface, including articular and calcified cartilage and excluding subchondral bone. The results obtained from selected ROIs from one microphotograph were averaged to obtain one data point representative of one experiment. For quantification, the selected ROI was converted to a contrasted image using color split plugin on ImageJ. The blue channel, which showed a contrasted image with clear view of the positive and negative signal, was selected to quantify signals. The images were converted to RGB, the positive signal was selected by threshold adjustment, also as described[4,5], and the total area constituting the positive signal was quantified. The fraction denoting positive signal in a given ROI was quantified by the formula: *Positive signal = (area of positive signal)/(total area of ROI)*

TUNEL assay:

We assessed chondrocyte apoptosis in WT (n=4) and cKO (n=6) mice at 12 weeks post-DMM using a TUNEL assay kit, following the manufacturer's instructions (Sigma, 11684795910 Roche, Darmstadt, Germany). Sections were coverslipped with ProLong Gold anti-fade mounting medium containing DAPI (ThermoFisher). Images were captured using a Nikon Eclipse Ni-E microscope. TUNEL-positive mean pixel density was measured and normalized to the DAPI+ signal, basically as described[2].

REFERENCES – SUPPLEMENTARY INFORMATION

1 Culley, K. L. *et al.* Mouse models of osteoarthritis: surgical model of posttraumatic osteoarthritis induced by destabilization of the medial meniscus. *Methods Mol Biol* **1226**, 143-173, doi:10.1007/978-1-4939-1619-1_12 (2015).

2 Wondimu, E. B. *et al.* Elf3 Contributes to Cartilage Degradation in vivo in a Surgical Model of Post-Traumatic Osteoarthritis. *Sci Rep* **8**, 6438, doi:10.1038/s41598-018-24695-3 (2018).

3 Dussault, A. A. & Pouliot, M. Rapid and simple comparison of messenger RNA levels using real-time PCR. *Biol Proced Online* **8**, 1-10, doi:10.1251/bpo114 (2006).

4 Girish, V. & Vijayalakshmi, A. Affordable image analysis using NIH Image/ImageJ. *Indian J Cancer* **41**, 47 (2004).

5 Varghese, F., Bukhari, A. B., Malhotra, R. & De, A. IHC Profiler: an open source plugin for the quantitative evaluation and automated scoring of immunohistochemistry images of human tissue samples. *PLoS One* **9**, e96801, doi:10.1371/journal.pone.0096801 (2014).

FIGURE LEGENDS – SUPPLEMENTARY INFORMATION

Supplementary Figure 1: ***Tamoxifen-inducible Cre recombinase activity in AcanCreER$^{T2/+}$;IKK$\alpha^{f/f}$ mice.*** Representative images of X-gal stained **(A)** hearts, **(B)** rib cages, and **(C)** hind paws harvested from vehicle- (WT) and tamoxifen-injected (cKO) mice showing specific LacZ staining in aggrecan-expressing tissues upon tamoxifen injection.

Supplementary Figure 2: ***Decreased Chuk mRNA in cKO mice at 8 weeks post-DMM.*** RTqPCR analyses of Chuk mRNA were performed in total RNA isolated from WT (n=4) and cKO (n=3) articular cartilage at 8 weeks post-DMM. *$p<0.05$ by *t*-test.

Supplementary Figure 3: ***Histological analyses of non-operated WT and cKO knee joints post-DMM.*** Representative Safranin-O/Fast green stained images of non-operated WT and cKO mice at **(A)** 8 (WT n=7, cKO n=8) and **(B)** 12 weeks (WT n=6, cKO n=6) after surgery DMM, in mice injected with vehicle or tamoxifen before surgery. Images of non-operated WT (n=10) and cKO (n=5) knee joints at 12 weeks after surgery in mice injected with vehicle or tamoxifen after surgery are shown in **(C)**. OARSI histological scores of non-operated WT and cKO mice (graphs on the right) showed no difference in proteoglycan content or structural integrity of the articular cartilage between groups. Scale bar = 100μm.

Supplementary Table 1: ***PCR primers and conditions***

GENE	Primer sequences	Size (bp)	Anneal (°C)	GenBank Accesion
Acan	Forward: 5'- GGTCACTGTTACCGCCACTT -3'<br>Reverse: 5'- CCCCTTCGATAGTCCTGTCA -3'	175	60	NM_007424.2
Col2a1	Forward: 5'- AATGGCACGGCTGTGTGCGA -3'<br>Reverse: 5'- AACGGGTCCCCTTGGGCCTT -3'	183	60	NM_031163.3
Col10a1	Forward: 5'- ACGCATCTCCCAGCACCAGAATC -3'<br>Reverse: 5'- GGGGCTAGCAAGTGGGCCCT -3'	148	60	NM_009925.4
Chuk	Forward: 5'-TCAAGATGTTGGTGGGAAGACA -3'<br>Reverse: 5'-AGCTCTGGGGCCAAATACTG -3'	120	60	NM_007700.2
Mmp2	Forward: 5'-TCGTGGCAGCCCATGAGTTCG-3'<br>Reverse: 5'-CATCGGGGGAGGGCCCATAGAG-3'	156	60	NM_008610.3
Mmp3	Forward: 5'-GTCCCTCTATGGAACTCCCACAGCA-3'<br>Reverse: 5'- GGACTTCTCCCCGGAGGGTGC-3'	140	60	NM_010809.2
Mmp10	Forward: 5'-AGCTGGCTGCTGTGCTGATCATCA-3'<br>Reverse: 5'-GAGCCACAGAACATGCAGGAGCA-3'	123	60	NM_019471.3
Mmp13	Forward: 5'-ATGGTCCAGGCGATGAAGACCCC-3'<br>Reverse: 5'-GTGCAGGCGCCAGAAGAATCTGT-3'	140	60	NM_008607.2
Runx2	Forward: 5'- TCCCCGGGAACCAAGAAGGCA -3'<br>Reverse: 5'- AGGGAGGGCCGTGGGTTCTG -3'	141	60	NM_001146038.2
Actb	Forward: 5'- GCCCTAGGCACCAGGGTGTGA -3'<br>Reverse: 5'- TCCTCAGGGGCCACACGCA -3'	190	60	NM_007393
Eef1a1	Forward: 5'- GCCTTGGTTCAAGGGATGGA -3'<br>Reverse: 5'- ACAGTGCCAATGCCTCCAAT -3'	162	60	NM_010106.2

Supplementary Figure 1:

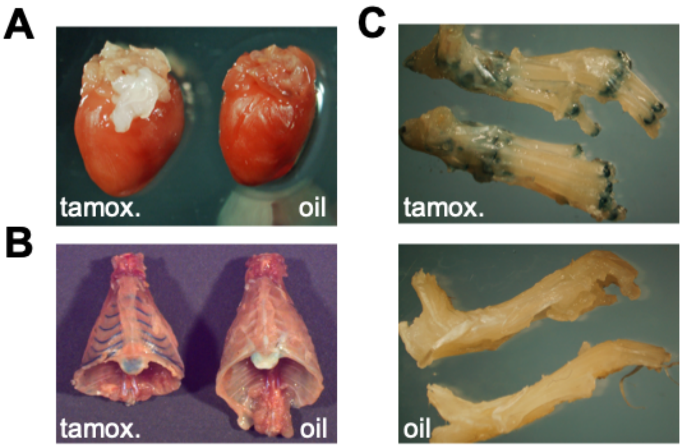

Supplementary Figure 2:

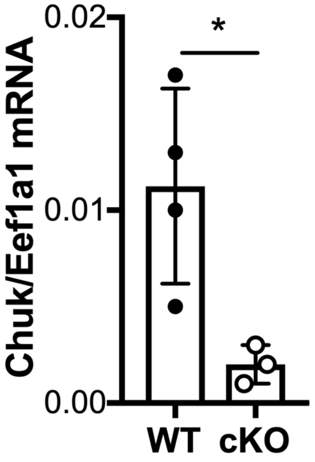

Supplementary Figure 3:

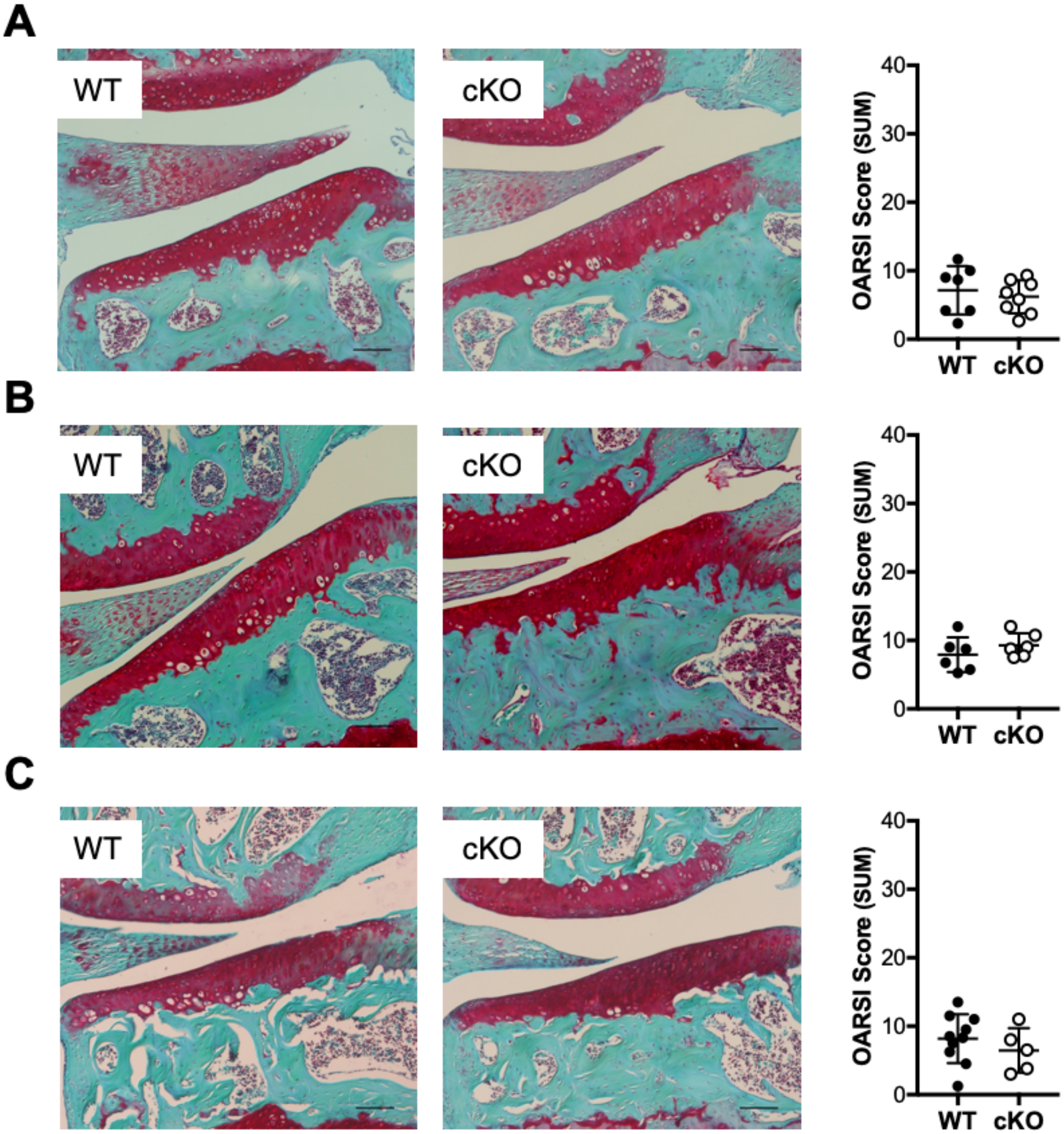

Supplement: Supplementary file 1 — Supplementary Information [file 41598_2019_45334_MOESM1_ESM.pdf]
